# Effect of Aminated Chitosan-Coated Fe_3_O_4_ Nanoparticles with Applicational Potential in Nanomedicine on DPPG, DSPC, and POPC Langmuir Monolayers as Cell Membrane Models

**DOI:** 10.3390/ijms22052467

**Published:** 2021-02-28

**Authors:** Emilia Piosik, Marta Ziegler-Borowska, Dorota Chełminiak-Dudkiewicz, Tomasz Martyński

**Affiliations:** 1 Faculty of Material Engineering and Technical Physics, Poznan University of Technology, Piotrowo 3, 60-965 Poznan, Poland; tomasz.martynski@put.poznan.pl; 2 Faculty of Chemistry, Nicolaus Copernicus University in Torun, Gagarina 7, 87-100 Torun, Poland; dorotachd@umk.pl

**Keywords:** magnetite nanoparticles, aminated chitosan, cell membrane, Langmuir film, nanomedicine

## Abstract

An adsorption process of magnetite nanoparticles functionalized with aminated chitosan (Fe_3_O_4_-AChit) showing application potential in nanomedicine into cell membrane models was studied. The cell membrane models were formed using a Langmuir technique from three selected phospholipids with different polar head-groups as well as length and carbon saturation of alkyl chains. The research presented in this work reveals the existence of membrane model composition-dependent regulation of phospholipid-nanoparticle interactions. The influence of the positively charged Fe_3_O_4_-AChit nanoparticles on a Langmuir film stability, phase state, and textures is much greater in the case of these formed by negatively charged 1,2-dipalmitoyl-*sn*-glycero-3-phospho-*ra*c-(1-glycerol) (DPPG) than those created by zwitterionic 1,2-distearoyl-sn-glycero-3-phosphocholine (DSPC) and 2-oleoyl-1-palmitoyl-*sn*-glycero-3-phosphocholine (POPC). The adsorption kinetics recorded during penetration experiments show that this effect is caused by the strongest adsorption of the investigated nanoparticles into the DPPG monolayer driven very likely by the electrostatic attraction. The differences in the adsorption strength of the Fe_3_O_4_-AChit nanoparticles into the Langmuir films formed by the phosphatidylcholines were also observed. The nanoparticles adsorbed more easily into more loosely packed POPC monolayer.

## 1. Introduction

Nanomaterials such as graphene, silicene, germanene, nanotubes, and nanoparticles thanks to their novel intrinsic properties have attracted the attention of researchers from both academic and industrial sectors in the chemical, environmental, optoelectronic, and medical fields [1,2,3,4,5,6,7,8,9,10,11,12,13,14,15,16,17]. An important place among them belongs to magnetite nanoparticles (Fe_3_O_4_), which show application potential, especially in nanomedicine in magnetic resonance imaging, hyperthermia, targeted drug delivery, enzyme immobilization, cell labeling, separation and purification of biomolecules, and in enrichment of DNA [18,19,20,21,22,23,24,25,26,27,28,29]. A small size, superparamagnetic properties, and wide chemical affinity of the magnetite nanoparticles stand behind their numerous possible applications in nanomedicine. However, the application of bare magnetite nanoparticles in the medical field is challenging because of their poor biocompatibility, the lack of biodegradability, and chemical instability in a physiological environment. These obstacles can be overcome by modification of a surface of nanoparticles using a method of ligand addition, ligand exchange, or encapsulation. The development in nanotechnology not only enables optimal bioperformance of magnetite nanoparticles, but also allows for tailoring of their surface in order to fine-tune their physicochemical properties for specific nanomedical applications [30,31].

For surface functionalization of the magnetite nanoparticles, both types of organic materials—low-molecular-mass compounds and polymers—can be employed. The magnetite nanoparticles with small-molecule ligands, e.g., citrates, phosphates, thiols, carboxylic acids, amines, and aminosilanes, in coronas are easy in preparation and characterized by simple conjugation chemistry [29]. Such coatings stabilize the nanoparticles, provide them a physical protection barrier, a small hydrodynamic radius required for nanomedical applications, and functional groups for linkage of biomolecules and drugs [24]. However, magnetite nanoparticles functionalized with polymers attract more attention thanks to their excellent colloidal stability. Polymer coatings increase repulsive forces balancing in this way magnetic and van der Waals attractive interactions occurring between the nanoparticles. Modification can take place using either natural or synthetic polymers [29]. The most widely studied natural polymers for coatings of magnetite nanoparticles are dextran, starch, and chitosan, while the synthetic ones include polyethylene glycol (PEG), polyvinylpyrrolidone (PVP), and polyvinyl alcohol (PVA) [29]. Chitosan plays among them a special role because it is a natural, biocompatible, biodegradable, and nontoxic polysaccharide. Moreover, many functional groups are present in its backbone structure. It has repeating units containing two hydroxyl groups and an amino group, which are highly reactive. Chitosan attached to the magnetite nanoparticles surface thanks to the presence of these groups allows to bind drugs and biomolecules, e.g., enzymes, proteins, and antibodies.

The knowledge of behavior of biomedical materials such as magnetite nanoparticles in contact with biological cells is of crucial importance. The majority of such kind of research is carried out on whole biological cells. However, this approach provides information only on an overall cell response and does not give an insight into the mechanism of action of biomaterials on the cell membrane at the molecular level. For this reason, the simplified experimental models of biological membranes such as lipid vesicles (liposomes) and planar lipid models (black lipid membranes, freely suspended layers, Langmuir monolayers) are often used by researchers as an extension of the studies involving living cells and tissues [32]. The most frequently used models of cell membranes are phospholipid Langmuir monolayers [33,34,35,36,37,38,39,40]. One of the reasons behind it is the fact that the Langmuir technique gives the possibility to design models of biological membranes with controlled composition and molecular packing density as well as environmental conditions such as temperature and pH [41,42]. This method is also free from limitations of the lipid vesicles model, e.g., providing mainly qualitative and visual data on transport across the membrane and its permeability.

In this work, the adsorption process of the magnetite nanoparticles functionalized with aminated chitosan (Fe_3_O_4_-AChit) into the cell membrane models made using the Langmuir technique at the air-water interface has been studied. Three amino groups long-distanced from the pyranose unit are attached to chitosan present in the corona of the Fe_3_O_4_-AChit nanoparticles. The presence of these additional amino groups improves their efficiency of binding of drugs and biomolecules, which increases their application potential in nanomedicine [43,44,45,46,47]. Previously, the interactions between the Fe_3_O_4_-AChit nanoparticles and the cell model membrane made of 1,2-dipalmitoyl-sn-glycero-3-phosphocholine (DPPC)—one of the most important phospholipids occurring in human cells—were examined [48]. Previous research has shown a strong influence of the investigated nanoparticles on the stability, phase state, and morphology of the DPPC membrane model. It was reported also that the Fe_3_O_4_-AChit nanoparticles can adsorb to the DPPC monolayer. Moreover, the number of the adsorbed/incorporated Fe_3_O_4_-AChit nanoparticles can be regulated by the nanoparticles concentration in the neighborhood of the DPPC membrane model even at the high surface pressure of 35 mN·m^−1^ (the value corresponding to a surface pressure occurring in natural cellular membranes) contrary to the magnetite nanoparticles coated with fatty acids [49]. These results indicate that in the case of the Fe_3_O_4_-AChit nanoparticles usage in drug delivery, the amount of drugs and biomolecules transferred to the human cell interior can be controlled by the number of the transported nanoparticles. Nevertheless, one should remember that the majority of natural membranes are a mixture of phospholipids and other components such as cholesterol and proteins. The differences in their composition are a result of the roles of particular cells in a human body and significantly affect membrane permeability, mechanical strength and biochemical interactions. The previous research provided an insight into an interaction mechanism between the Fe_3_O_4_-AChit nanoparticles and DPPC. However, there is still the lack of the knowledge of the interactions of the investigated nanoparticles with other phospholipids present in the native cell membranes. For these reasons, the presented research is devoted to the adsorption process of the Fe_3_O_4_-AChit nanoparticles into membrane models made of phospholipids with different polar head-groups as well as length and carbon saturation of alkyl chains such as:

- 1,2-distearoyl-sn-glycero-3-phosphocholine (DSPC),
- 1,2-dipalmitoyl-sn-glycero-3-phospho-rac-(1-glycerol) (DPPG), and
- 2-oleoyl-1-palmitoyl-sn-glycero-3-phosphocholine (POPC).

## 2. Results and Discussion

**Figure 1:**
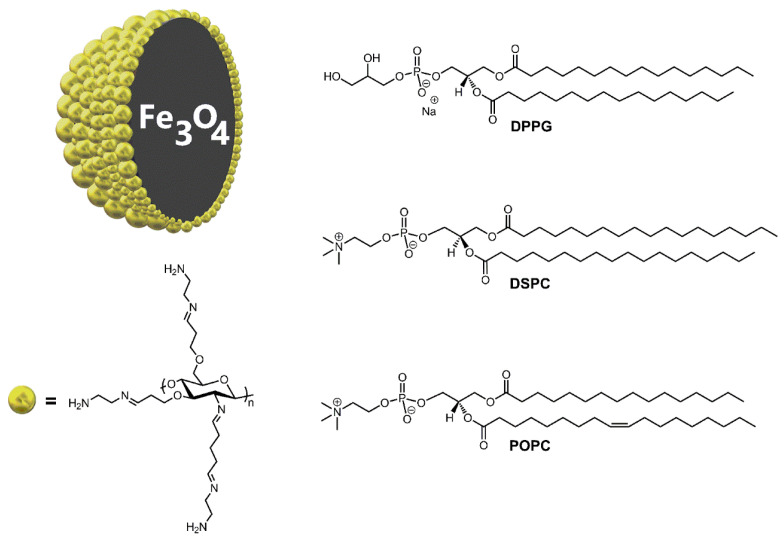
Molecular structure of the Fe_3_O_4_-AChit nanoparticles, 1,2-dipalmitoyl-sn-glycero-3-phospho-rac-(1-glycerol) (DPPG), 1,2-distearoyl-sn-glycero-3-phosphocholine (DSPC), and 2-oleoyl-1-palmitoyl-sn-glycero-3-phosphocholine (POPC).

In order to assess the effect of the Fe_3_O_4_-AChit nanoparticles (Figure 1) on the DPPG, DSPC, and POPC monolayers constituting the cell membrane models, two types of experiments were performed. In the first one, the phospholipid Langmuir films were compressed on the water subphase with the different concentrations of the Fe_3_O_4_-AChit nanoparticles. During their compression, surface pressure (*π*)-mean molecular area per phospholipid molecule (*A*) isotherms (*π-A* isotherms) and Brewster angle microscope (BAM) images were recorded. Their analysis provides information on the influence of the Fe_3_O_4_-AChit nanoparticles on a thermodynamic state, phase transitions, and morphology of the phospholipid monolayers. The *π-A* isotherms, *C*_S_^−1^-*π* dependences, and BAM images recorded during compression of the DPPG, DSPC, and POPC monolayers on the pure water subphase with the different concentrations of the Fe_3_O_4_-AChit nanoparticles are presented and discussed in Section 2.1.

The second type of performed experiment was a penetration experiment, which delivered detailed information on the adsorption process of the Fe_3_O_4_-AChit nanoparticles into the DPPG, DSPC, and POPC monolayers. The adsorption kinetics recorded during the penetration experiments are shown and discussed in Section 2.2.

### 2.1. Phospholipid Films Compressed on the Water Subphase Containing the Fe_3_O_4_-AChit Nanoparticles

#### 2.1.1. DPPG Compressed on the Water Subphases with the Different Concentrations of the Fe_3_O_4_-AChit Nanoparticles

**Figure 2:**
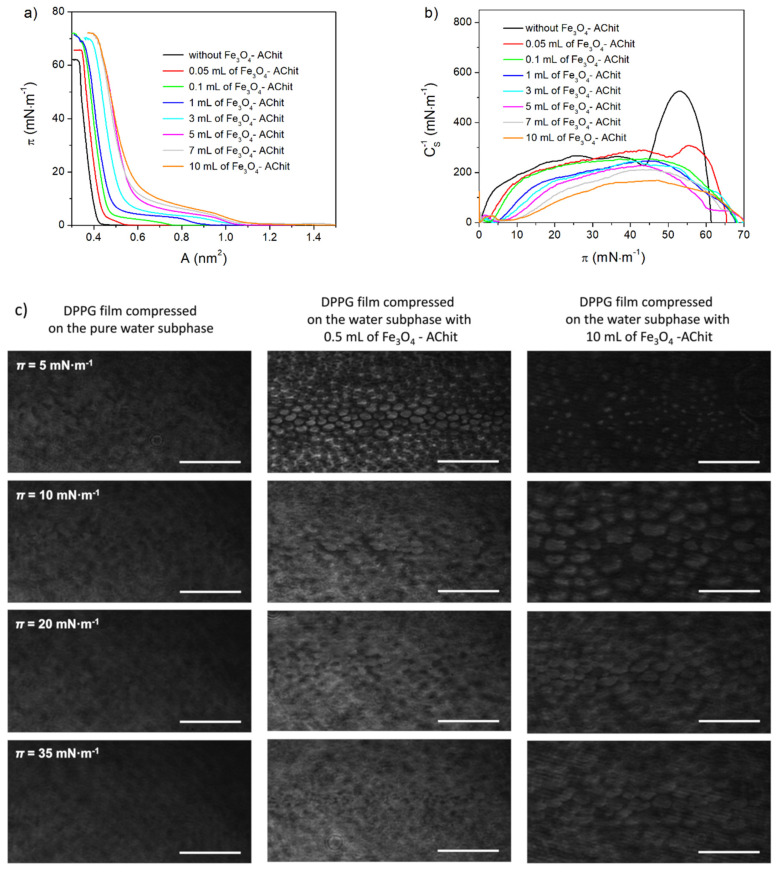
π-A isotherms (**a**), C_S_^−1^-π dependences (**b**), and Brewster angle microscope (BAM) images (**c**) recorded during compression of the DPPG monolayers on the water subphase with different concentrations of the Fe_3_O_4_-AChit nanoparticles. Length of scale bar in BAM images—100 μm.

DPPG is the saturated phospholipid formed of the polar head-group containing phosphatidylglycerol and two hydrocarbon chains comprising 16 carbon atoms (Figure 1). In Figure 2, the *π-A* isotherms, *C*_S_^−1^-*π* dependences, and BAM images recorded during compression of DPPG on the pure and containing different concentrations of Fe_3_O_4_-AChit nanoparticles water subphase are presented. At the beginning of the DPPG compression on the water without the nanoparticles at *π* = 0 mN·m^−1^ (Figure 2a, black line), the pure fluid (gaseous) phase does not occur and the monolayer exists in the two-phase coexistence state. The two-dimensional condensation of DPPG starts at *A* ≈ 1 nm^2^ as it is known from the literature [50]. The surface pressure increase, associated with the transition to the liquid condensed phase, begins at an extrapolated mean molecular area *A*_EXT_ ≈ 0.41 nm^2^ (*A*_EXT_ value is determined by the extrapolation of a tangent of a first linear slope of the isotherms to *π* = 0). Further compression of the DPPG monolayer causes the appearance of the phase transition to the solid state, which takes place at *A* = 0.35 nm^2^ and *π* = 43.6 mN·m^−1^. The *C*_S_^−1^ value increases after this transition up to ≈ 530 mN·m^−1^. It exhibits very close packing of the DPPG molecules at this experiment stage. The collapse of the DPPG film occurs at *A* = 0.33 nm^2^ and *π* = 61.8 mN·m^−1^. During the whole compression process of DPPG, the BAM images show irregularly shaped textures characteristic for films with changing molecular packing density in their different regions (Figure 2c). The isotherm and BAM images recorded for the DPPG compressed on the pure water subphase are consistent with those presented in the literature [50,51,52].

The Fe_3_O_4_-AChit nanoparticles present in the subphase strongly influence the course of the compression isotherms of the DPPG films (Figure 2a). The *π-A* isotherms recorded for the DPPG monolayers compressed on the water containing the nanoparticles are shifted in the direction of the larger areas per molecule. This shift and thus the film expansion increases with the rising concentration of the Fe_3_O_4_-AChit nanoparticles. It can indicate the adsorption of the investigated nanoparticles into the phospholipid monolayers. The presence of the Fe_3_O_4_-AChit nanoparticles in the water also causes the appearance of the liquid expanded phase between the gaseous state and liquid condensed phase. The horizontal plateau region representing the first-order transition from the liquid expanded state to the liquid condensed phase appears in the *π-A* isotherms starting from that recorded for the DPPG monolayer compressed on the subphase containing the volume of 1 mL of the nanoparticles’ suspension. When the concentration of the Fe_3_O_4_-AChit nanoparticles in the subphase increases, the plateau region becomes shorter and is no longer perfectly horizontal. Furthermore, the liquid expanded-liquid condensed phase transition occurs at the higher and higher π values (Figure 2a,b). In the BAM images taken near this phase transition (Figure 2c), the ellipsoidal domains of DPPG in the liquid condensed phase with the monolayer in the liquid expanded phase as a background are visible. Such domains were not observed during the compression of the DPPG on pure water. The BAM observations showed also the increase of the surface pressure value of the formation of the first visible liquid condensed domains (data not shown) and distances between them together with the concentration of the nanoparticles. This suggests that the Fe_3_O_4_-AChit nanoparticles probably are located preferably into loosen monolayer areas in the liquid expanded phase. A similar behavior was reported by Matshaya et al. [53] for the magnetite nanoparticles stabilized with oleic acid in contact with the DPPC Langmuir monolayer.

The adsorbed Fe_3_O_4_-AChit nanoparticles hinder the coalescence of the liquid condensed DPPG domains into a homogeneous monolayer when the plateau ends. In the case of the DPPG monolayer compressed on the subphase containing 10 mL of the Fe_3_O_4_-AChit suspension, these domains are still visible at *π* = 35 mN·m^−1^ and the DPPG molecules do not create perfectly homogeneous films even just before its collapse. The shift of the *π-A* isotherms obtained for the DPPG films formed on the subphase with the nanoparticles to the larger areas per molecule is observed even at the high surface pressure values (*π* > 10 mN·m^−1^) and the collapse surface pressure grows with nanoparticles’ concentration (see Figure 2a). Therefore, in the most compressed state, the expansion of the DPPG films occurs and the Fe_3_O_4_-AChit nanoparticles remain adsorbed into them. It is also worth noticing that the presence of the Fe_3_O_4_-AChit nanoparticles causes the decrease of the maximal *C*_S_^−1^ value associated with the disappearance of the solid state at the DPPG final compression stage.

#### 2.1.2. DSPC Compressed on the Water Subphases with the Different Concentrations of the Fe_3_O_4_-AChit Nanoparticles

**Figure 3:**
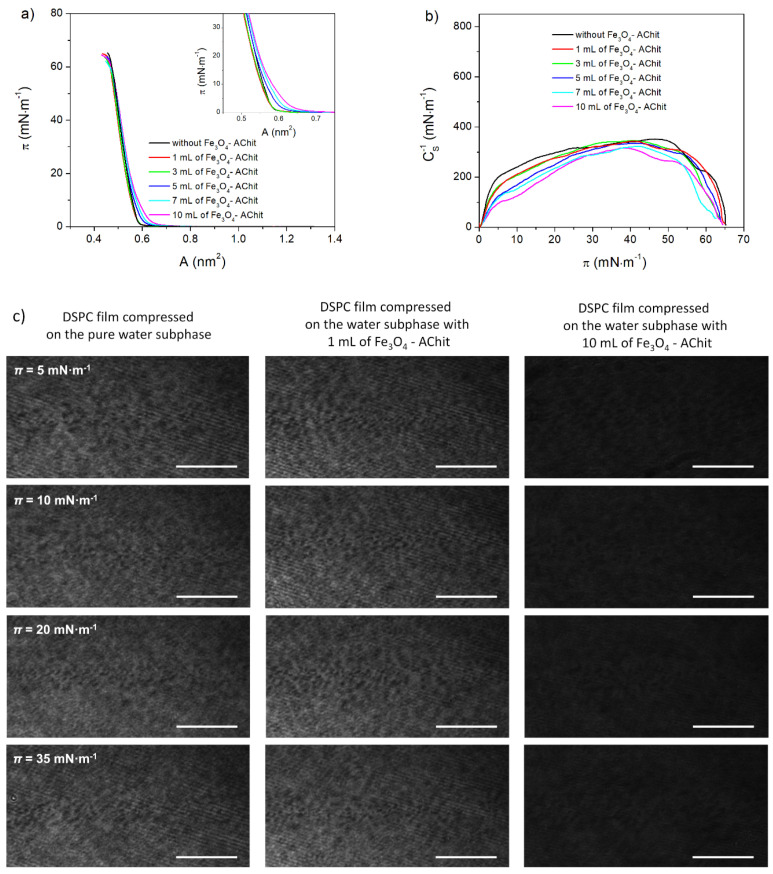
π-A isotherms (**a**), C_S_^−1^-π dependences (**b**), and BAM images (**c**) recorded during compression of the DSPC monolayers on the water subphase with different concentrations of the Fe_3_O_4_-AChit nanoparticles. Length of scale bar in BAM images—100 μm.

DSPC has phosphatidylcholine in the polar head-group instead of phosphatidylglycerol like it takes place in the case of DPPG (Figure 1). Its hydrocarbon chains are composed of 18 carbon atoms. One can see in Figure 3a (black line) that the surface pressure increase associated with the transition of DSPC compressed on the pure water subphase from the gaseous phase to the condensed state starts at the extrapolated mean molecular area *A*_EXT_ ≈ 0.57 nm^2^. The *A*_EXT_ value determined for DSPC monolayer is thus larger by 0.19 nm^2^ than in the case of DPPG film. This exhibits smaller molecular packing density at the initial compression stage in the DSPC Langmuir film than in the DPPG one. The *π-A* isotherm of DSPC is very steep in the region of the surface pressure increase, which indicates the chain-ordered state. In the *C*_S_^−1^-*π* dependence course, no minima characteristic for the phase transitions is observed before the monolayer collapse. Furthermore, *C*_S_^−1^ reaches the value of 353 mN·m^−1^ corresponding to the solid state (Figure 3b). This all shows that the surface pressure increase observed in the *π-A* isotherm is related to the rapid growth of the molecular packing density in the DSPC film and the transition to the solid state. The maximal *C*_S_^−1^ value determined for the DSPC monolayer is smaller by 292 mN·m^−1^ than that evaluated for DPPG. Therefore, also at the final compression stage, the DSPC film is significantly more rigid and the molecules within it are less densely packed. In the BAM images recorded during the whole compression process of the DSPC monolayer (Figure 3c), the areas with different grey shades and an irregular shape representing the regions of the film characterized by the different molecular packing densities are visible. The results obtained for DSPC compressed on the pure water subphase agree with those presented in the literature [54,55].

The presence of the Fe_3_O_4_-AChit nanoparticles in the water subphase does not cause a significant shift of the DSPC compression isotherms in the direction of the larger mean molecular areas like it takes place in the case of DPPG (Figure 3a). The change of their shape is observed at the initial stage of the surface pressure increase (Figure 3a, inset). However, they practically coincide at the high surface pressures. The maximal *C*_S_^−1^ value does not decrease significantly under the influence of the Fe_3_O_4_-AChit nanoparticles, which means that the phase state of the DSPC monolayer remains unchanged (Figure 3b). The presence of the nanoparticles in the subphase also does not change the DSPC film texture in the range of the investigated concentrations. It is visible in the recorded BAM images (Figure 3c). All these findings analyzed together indicate that the single Fe_3_O_4_-AChit nanoparticles can perhaps adsorb very weakly to the DSPC monolayer at the beginning of the compression. However, further compression leads to the reduction of the molecular area and to almost total squeezing them out of the DSPC monolayer.

Due to the smaller packing density and thus a weaker van der Waals interaction between phospholipid molecules in the DSPC monolayer than in the DPPG film, the Fe_3_O_4_-AChit nanoparticles should adsorb more easily into the former one. However, one can notice analyzing the *π-A* isotherms and BAM images that the effect of the Fe_3_O_4_-AChit nanoparticles on the DSPC monolayers is much smaller than their influence on the DPPG films. The main reason behind it seems to be a stronger electrostatic attraction between the Fe_3_O_4_-AChit nanoparticles and DPPG molecules. The Fe_3_O_4_-AChit nanoparticles are functionalized with the aminated chitosan, which is positively charged. Therefore, their electrostatic attraction to the monolayers created by the DPPG molecules possessing the negatively charged polar head-groups is stronger than to those formed by the DSPC molecules with the zwitterionic head-groups. A similar effect was observed by Uehara et al. during the studies on the adsorption process of the magnetite nanoparticles stabilized with poly(diallydimethylammonium chloride) (Fe_3_O_4_-PDAC) and dextran (Fe_3_O_4_-Dext) from the water subphase to the Langmuir phospholipid membranes formed by DPPG and DPPC [56]. The research performed by Uehara et al. showed that the positively charged Fe_3_O_4_-PDAC nanoparticles strongly adsorb into the DPPG monolayer. In contrast, the negatively charged Fe_3_O_4_-Dext nanoparticles remain in water due to the electrostatic repulsion between them and the DPPG molecules. Both types of the magnetite nanoparticles—Fe_3_O_4_-PDAC and Fe_3_O_4_-Dext—are capable of interacting with zwitterionic DPPC. Furthermore, the studies of Torrano et al. revealed that the interactions of the oppositely charged gold nanoparticles modified with citrate anions or cationic polyelectrolyte poly(allylamine hydrochloride) with the DPPG and DPPC Langmuir monolayers are dominated by the electrostatic effects [52]. These effects also govern binding of the anionic and cationic silver nanoparticles coated, respectively, with polyethylenimine (Ag−NH) and with a carboxylated amphiphilic polymer (Ag−COOH) to net-anionic PC/PG monolayers formed from the saturated or unsaturated lipids. Bothun et al. showed that the anionic Ag−COOH nanoparticles incorporate into saturated DPPC/DPPG and unsaturated DOPC/DOPG monolayers at the low initial surface pressure (10 mN·m^−1^) and cause phospholipid condensation at high initial surface pressures (20 and 30 mN·m^−1^) [57]. The responsibility for incorporation was ascribed to hydrophobic interactions, but the responsibility for phospholipid condensation was assigned to the electrostatic and charge-dipole interactions with PCs. On the other hand, the Ag−NH nanoparticles incorporate only into the saturated DPPC/DPPG monolayers, which leads to lipid condensation driven primarily by the electrostatic interactions with PGs. Previous studies on the magnetite, gold, and silver nanoparticles showed the importance of electrostatic forces in the nanoparticles-membrane model interactions. However, in the case of the Fe_3_O_4_-AChit nanoparticles, attention should also be paid to the hydrophilic nature of the aminated chitosan surface. Based on the measurement of the glycerol contact angle for this material (87°), it can be concluded that it easily interacts with glycerine, which in its structure corresponds to the part of the “head” of DPPG [47].

A comparison of previous research related to the adsorption of the Fe_3_O_4_-AChit nanoparticles into the DPPC monolayer [48] to the current studies on their interactions with DSPC can be beneficial when it comes to the evaluation of the influence of the hydrocarbon chains length of the phospholipids. This comparison shows the greater effect of the Fe_3_O_4_-AChit nanoparticles present in the water subphase on the DPPC films than on the DSPC ones. The *π-A* isotherms recorded for the DPPC are shifted towards the larger mean molecular areas, but the shape and thus phase behavior of DPPC is not as significantly affected as for DPPG. Nevertheless, the interactions of the Fe_3_O_4_-AChit nanoparticles with DPPC are stronger than with DSPC and their adsorption into the DPPC monolayers was postulated. The DPPC isotherms not coincide even at high surface pressures. This means that the nanoparticles are present in the monolayers until the end of compression and are not squeezed out of the film contrary to the case of DSPC. DSPC possess longer alkyl chains compared to DPPC. The van der Waals interaction between the molecules of phospholipids with the longer hydrocarbon chains is stronger and their molecules create the more densely packed monolayers at the air-water interface [58]. Indeed, the *A*_EXT_ value determined for DSPC is almost two times larger than that obtained for DPPC. Therefore, the DSPC molecules are more densely packed than DPPC ones already at the beginning compression stage. The maximal *C*_S_^−1^ is also higher in the case of DSPC by ≈ 100 mN·m^−1^. Furthermore, at the final compression stage, the DSPC monolayer is in the solid state, while the DPPC film is in the liquid condensed phase. The greater molecular packing density in the case of the DSPC explains thus more difficult adsorption of the nanoparticles into its monolayer than into the DPPC film.

#### 2.1.3. POPC Compressed on the Water Subphases with the Different Concentrations of the Fe_3_O_4_-AChit Nanoparticles

**Figure 4:**
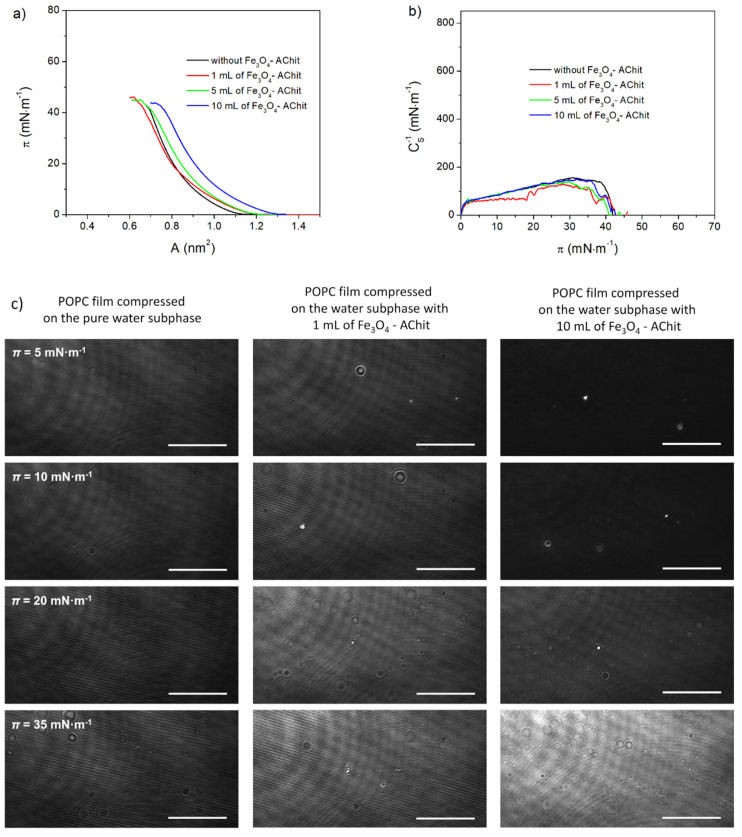
π-A isotherms (**a**), C_S_^−1^-π dependences (**b**), and BAM images (**c**) recorded during compression of the POPC monolayers on the water subphase with different concentrations of the Fe_3_O_4_-AChit nanoparticles. Length of scale bar in BAM images—100 μm.

POPC is an unsaturated phospholipid with phosphatidylcholine in the polar head-group and two hydrocarbon chains comprising 16 and 18 carbon atoms (Figure 1). The isotherm recorded during compression of POPC on the pure water subphase is shown in Figure 4a (black line). This isotherm is consistent with those presented in the literature [59,60]. DSPC creates the relatively loosely packed Langmuir monolayers, which is characteristic for unsaturated phospholipids. The increase of the surface pressure during its compression starts at *A*_EXT_ = 1.09 nm^2^. Maximal *C*_S_^−1^ is relatively low and reaches the value of 154 mN·m^−1^. This value corresponds to the liquid condensed phase (Figure 4b). The recorded BAM images show that the POPC monolayer is homogeneous during the whole compression process (Figure 4c).

The *π-A* isotherms of the POPC films compressed on the subphase containing the Fe_3_O_4_-AChit nanoparticles are shifted in the direction of the larger mean molecular areas in comparison to that recorded for POPC monolayer formed on the pure water (Figure 4a). They are also less steep and *C*_S_^−1^ values are slightly lower. This indicates a lower stability of the POPC films in the presence of the Fe_3_O_4_-AChit nanoparticles (Figure 4b). The shift of the isotherms recorded in the presence of the nanoparticles was also observed in the area of the high surface pressure values indicating that the nanoparticles remain adsorbed at this compression stage. In the BAM images, the small circular domains are visible (Figure 4c). These domains are probably formed by the nanoparticles adsorbed into the phospholipid monolayer. The formation of the domains with similar size and shape by the magnetite nanoparticles modified with oleic acid in the POPC film was reported by Matshaya et al. [53]. The changes in the course of the compression isotherms and morphology of the POPC monolayers under the influence of the Fe_3_O_4_-AChit nanoparticles are greater than in the case of DSPC. POPC creates significantly more loosely packed Langmuir films comparing to DSPC and this can facilitate the adsorption of the nanoparticles.

### 2.2. Adsorption Kinetics of the Fe_3_O_4_-AChit Nanoparticles to the Phospholipid Monolayers

**Figure 5:**
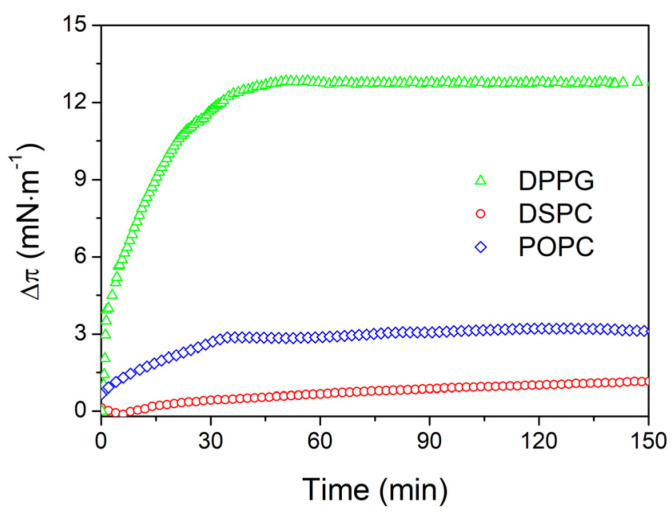
Kinetics of the adsorption process of the Fe_3_O_4_-AChit nanoparticles from the water subphase into the phospholipid membrane models recorded at the surface pressure equal to π = 35 mN·m^−1^.

The adsorption kinetics of the Fe_3_O_4_-AChit nanoparticles into the DPPG, DSPC, and POPC monolayers are presented in Figure 5. In all the cases, the increase of Δπ was observed after the injection of the Fe_3_O_4_-AChit suspension into the water subphase. This indicates the adsorption of the nanoparticles into the membrane models formed by all the phospholipids used in research. Furthermore, it is clearly seen that the most significant increase of Δπ and the strongest adsorption of the positively charged Fe_3_O_4_-AChit nanoparticles took place into the negatively charged DPPG membrane. The adsorption process was much weaker when the monolayers were formed from the zwitterionic phospholipids possessing attached phosphatidylcholine to the polar head-group—DPPC (previous studies [48]), DSPC, and POPC. It shows that the penetration experiments are in good agreement with the *π-A* isotherms and BAM images recorded during compression of the investigated phospholipids on the subphase containing the Fe_3_O_4_-AChit nanoparticles. The latter ones showed the greatest influence of the Fe_3_O_4_-AChit nanoparticles on the stability, phase state, and textures of the DPPG monolayers among all the investigated phospholipid films. The analysis of the adsorption kinetics seems to confirm that this influence is related to the strongest adsorption of the Fe_3_O_4_-AChit nanoparticles into the DPPG Langmuir film driven probably mainly by the electrostatic attraction.

The adsorption kinetics allowed to make a precise comparison of the strength of the adsorption of the Fe_3_O_4_-AChit nanoparticles into the Langmuir monolayers formed by the phosphatidylcholines. This was not possible based only on the *π-A* isotherms and BAM images recorded during compression of the phosphatidylcholines on the subphase containing the nanoparticles. The penetration experiments showed that the adsorption process is stronger in the case of the POPC than in the case of the DPPC (previous studies [48]) and DSPC films. The differences in the strength of the adsorption of the investigated nanoparticles into the monolayers formed by the particular phosphatidylcholines are a result of the different packing densities of their molecules. POPC creates significantly loosely packed Langmuir films than DPPC and DSPC, which can explain the stronger adsorption of the nanoparticles into its monolayer and its greater influence on the *π-A* isotherms and BAM images. Moreover, POPC contains in its structure a double bond. As it is known, *p*-type bonds have a partial negative charge associated with the electrons present on the unhybridized *p*-type orbits. This can additionally enhance the zwitterionic phospholipid-positively charged aminated chitosan interaction.

## 3. Materials and Methods

### 3.1. Materials

#### 3.1.1. Synthesis and Characterization of the Fe_3_O_4_-AChit Nanoparticles

The synthesis procedure of the Fe_3_O_4_-AChit nanoparticles and their physicochemical characterization were described in detail elsewhere [43,44,45,46,47,48,61,62,63]. Here, only the most important results of this research are presented. At the beginning of the Fe_3_O_4_-AChit nanoparticles synthesis, chitosan 0.1 g was mechanically stirred at room temperature in 1% acetic acid solution (10 mL) for 20 min. Then, iron (II) chloride tetrahydrate (0.37 g, 1.88 mmol) and iron (III) chloride hexahydrate (1.01 g, 3.75 mmol) were added (1:2 molar ratio) and the resulting solution was chemically precipitated at room temperature by adding dropwise 30% solution of NaOH (3.5 mL). The black mixture was formed immediately and epichlorohydrin (0.1 mL, 1.25 mmol) was added. Mixture was stirred at 50 °C for 2 h. After cooling to room temperature, sodium periodate solution (0.08 g in 1.25 mL of water) was added and the mixture was stirred for 30 min. The black precipitate was separated by filtration and washed by deionized water. Next, 5 mL of bicarbonate buffer pH—10 and 5 mL 5% glutaraldehyde solution was added, and the mixture was mechanically stirred at room temperature for 1 h, separated by magnet, and dried under vacuum. Obtained magnetic nanoparticles were pounded with ethylenediamine (1.2 g, 20 mmol) in an agate mortar at room temperature for 1 min without solvent. The resulting magnetic material was washed five times with deionized water and dried by lyophilization.

X-ray diffraction showed that the core of the investigated nanoparticles is made of pure Fe_3_O_4_ with a spinel structure. A successful generation of the long-distanced NH_2_ groups on the surface of the nanoparticles was confirmed by attenuated total reflectance Fourier transform infrared (Perkin Elmer, Waltham, MA, USA) analysis. Dynamic light scattering (Malvern Nano Zetasizer ZS90 instrument, Malvern, Malvern, UK) and transmission electron microscopy (Tecnai F20 X-Twin, FEI Europe) revealed that the synthesized Fe_3_O_4_-AChit nanoparticles are of the uniform size of 29 nm. Their magnetization hysteresis curves recorded using a superconducting quantum interference device (model MPMS3, San Diego, CA, USA) possess shape characteristic for the superparamagnetic materials and reduced from −77 emu/g to −30 emu/g saturation magnetization in comparison to the naked magnetite nanoparticles.

#### 3.1.2. Phospholipids

All the phospholipids used were purchased as a powder from Avanti Polar Lipids (Alabaster, Alabama, USA) and dissolved in spectroscopically pure chloroform (Avantor, Gliwice, Poland) at the concentration of 1 mM to obtain stock solutions.

### 3.2. Methods

#### 3.2.1. Preparation of the Water Suspensions of the Fe_3_O_4_-AChit Nanoparticles

Water suspensions of the Fe_3_O_4_-AChit nanoparticles used in the Langmuir monolayer experiments were prepared according to the following procedure: dispersed in the deionized water from the Milli-Q system (Merck, Darmstadt, Germany) at the concentration of 1 mg·mL^−1^, kept in an ultrasonic washer for 1 h, and filtered through nylon syringe filters with the pore diameter of 200 nm. Their final concentrations measured using a quartz crystal microbalance (openQCM, Pompeii, Italy) were equal to 0.40 ± 0.05 mg·mL^−1^.

#### 3.2.2. Compression of the Phospholipid Monolayers in the Subphase Containing the Fe_3_O_4_-AChit Nanoparticles

In order to study the interactions of the Fe_3_O_4_-AChit nanoparticles and phospholipid monolayers constituting biological membrane models, a hydrophobic Teflon Langmuir through (Minitrough 2 from KSV NIMA, Helsinki, Finland) was used. The phospholipid monolayers were compressed using two symmetrically driven hydrophilic barriers. The surface pressure measurement during compression was carried out with the accuracy of ± 0.1 mN·m^−1^ employing a platinum Wilhelmy plate with a highly porous surface hooked on an electronic balance. Ultrapure deionized water (250 mL) with the final resistivity of 18.2 MΩ·cm from the Milli-Q system containing the magnetite nanoparticles was used as a subphase. The concentration of the nanoparticles in the subphase was in the range of 0.15–15 μg·mL^−1^. The change of the water subphase pH after adding the Fe_3_O_4_-AChit suspension was not observed. Its constant temperature of 20 °C was kept by a cooling circulator (F12-ED from Julabo, Seelbach, Germany). The phospholipid chloroform solutions were deposited dropwise at the air-water interface with a glass microliter syringe (Hamilton, Bonaduz, Switzerland). The compression of the phospholipid Langmuir monolayers took place at the barrier motion speed of 2 mm·min^−1^ after 30 min for solvent evaporation. During this process, the *π-A* isotherms were recorded. All measurements were repeated on the fresh subphase three times to confirm reproducibility. Based on the *π-A* isotherms, dependences of a compression modulus (*C*_S_^−1^) in a function of the surface’s pressure were determined according to the following equation [41,42]:

C_S_^−1^ = −A δπ/δA. (1)

Phase transition points are visible in *C*_S_^−1^-*π* plots as characteristic minima. This allows to estimate precisely the surface pressure values at which transitions occur. Moreover, the *C*_S_^−1^ delivers further information on the phase state of the Langmuir films. In order to classify the phase state of the investigated phospholipid monolayers, the Davies and Rideal criterion was used [64]. According to this criterion, *C*_S_^−1^ is in the range of 12.5–50 mN·m^−1^ for a liquid expanded (LE) phase, 50–100m N·m^−1^ for a liquid state (L), 100–250 mN·m^−1^ for a liquid condensed (LC) phase, and > 250 mN·m^−1^ for a solid state.

#### 3.2.3. Brewster Angle Microscopy Observations

The textures of the phospholipid monolayers compressed on the water with the different concentrations of the Fe_3_O_4_-AChit nanoparticles were visualized in situ using a custom-made Brewster angle microscope (BAM) constructed on the basis of the Moebius set-up [65,66]. The lateral resolution of used BAM is ≈2 μm. Only a horizontally placed strip in the center of the presented BAM images is in focus.

#### 3.2.4. Penetration Experiments

In order to record the kinetics of the adsorption process, the phospholipid monolayers were compressed on the pure water subphase until the surface pressure value of 35 mN·m^−1^ was reached. Subsequently, the volume of 5 mL of the Fe_3_O_4_-AChit water suspension was injected into the water subphase behind the barriers in order to avoid disruption of the monolayer and the surface pressure was monitored at the fixed barrier position (*π*^Phospholipid + Fe^_3_^O^_4_^−AChit^ (*t*)). The reference experiment was performed at the same time and under identical experimental conditions. In the reference experiment, the phospholipid monolayer was also compressed to *π* = 35 mN·m^−1^ on the pure water surface, but the surface pressure (*π*^Phospholipid^(*t*)) was monitored without injecting of the Fe_3_O_4_-AChit suspension into the subphase. The change of the surface pressure under an influence of the nanoparticles present in the water subphase (Δ*π*) was calculated as follows:

Δ*π = π*^Phospholipid + Fe^_3_^O^_4_^−AChit^(*t*) − *π*^Phospholipid^(*t*). (2)

Such an approach allows eliminating the water evaporation effect from the Langmuir trough and even small fluctuations of the external conditions, e.g., temperature or humidity, on the time-consuming experiments. The increase of the Δπ value means the insertion of the nanoparticles into the phospholipid monolayer, while its decrease is associated with the condensation of the phospholipid molecules on the nanoparticles due to nanoparticles-phospholipid binding or their extraction from the air-water interface and the subsequent sink into the water subphase [49,57,67]. The penetration experiments were performed on the same setup and at the same experimental conditions like it took place in the case of the phospholipid compression on the subphase containing the Fe_3_O_4_-AChit nanoparticles.

## 4. Conclusions

In conclusion, the adsorption process of the aminated chitosan-coated magnetite nanoparticles (Fe_3_O_4_-AChit nanoparticles) with application potential in nanomedicine into the phospholipid Langmuir monolayers made of DPPG, DSPC, and POPC constituting the cell membrane models was investigated. The *π-A* isotherms and BAM images showed that the influence of the Fe_3_O_4_-AChit nanoparticles on the stability, phase behavior, and textures of the phospholipid Langmuir films was much greater in the case of those formed by DPPG than those created by DPPC (previous research [48]), DSPC, and POPC. Moreover, the adsorption kinetics recorded during penetration experiments revealed the strongest adsorption of the Fe_3_O_4_-AChit nanoparticles into the DPPG monolayer. The differences in an interaction strength of the Fe_3_O_4_-AChit nanoparticles with the monolayers made of DPPG representing the phosphatidylglycerols and those formed by the phosphatidylcholines are caused presumably by the stronger electrostatic attraction of the positively charged nanoparticles to the negatively charged DPPG molecules than to the zwitterionic DPPC, DSPC, or POPC molecules. The penetration experiments also showed a different strength of the adsorption of the Fe_3_O_4_-AChit nanoparticles into the films formed by the phosphatidylcholines used in the current and previous research [48]. POPC creates the most loosely packed monolayers among investigated phosphatidylcholines, while DSPC the most densely packed. It makes that the Fe_3_O_4_-AChit nanoparticles adsorb the most easily into the POPC monolayers and the most weakly into the DSPC films. The second factor playing an important role in the adsorption process of the Fe_3_O_4_-AChit nanoparticles into the model membranes after the electrostatic interactions seems to be thus their molecular packing density determined by the length and saturation degree of the phospholipid hydrocarbon chains. Although performed on biological membrane models, the presented research provides an insight into the interaction mechanism of the magnetic nanoparticles and biological cells. The obtained results indicate that the Fe_3_O_4_-AChit nanoparticles can adsorb more easily into the bacterial cell membranes containing ~25% negatively charged lipids than into the mammalian cell membrane usually consisting of the zwitterionic lipids such as the phosphatidylcholines. This knowledge can be useful in future studies on their possible application in nanomedicine.

## Data Availability

Not applicable.
